# Exploring the Influence of Item Characteristics in a Spatial Reasoning Task

**DOI:** 10.3390/jintelligence11080152

**Published:** 2023-07-31

**Authors:** Qingzhou Shi, Stefanie A. Wind, Joni M. Lakin

**Affiliations:** Department of Educational Studies in Psychology, Research Methodology, and Counseling, College of Education, University of Alabama, Tuscaloosa, AL 35487, USA; stefanie.wind@ua.edu (S.A.W.); jlakin@ua.edu (J.M.L.)

**Keywords:** spatial reasoning, visuospatial ability, linear logistic test models, explanatory response models, item response theory

## Abstract

Well-designed spatial assessments can incorporate multiple sources of complexity that reflect important aspects of spatial reasoning. When these aspects are systematically included in spatial reasoning items, researchers can use psychometric models to examine the impact of each aspect on item difficulty. These methods can then help the researchers to understand the nature and development of spatial reasoning and can also inform the development of new items to better reflect the construct. This study investigated sources of item difficulty for object assembly (OA), a format for the assessment of spatial reasoning, by specifying nine item characteristics that were predicted to contribute to item difficulty. We used data from two focal samples including high-ability students in grades 3 to 7 and undergraduate students who responded to 15 newly developed OA items. Results from the linear logistic test model (LLTM) indicated that eight of the nine identified item characteristics significantly contributed to item difficulty. This suggests that an LLTM approach is useful in examining the contributions of various aspects of spatial reasoning to item difficulty and informing item development for spatial reasoning assessments.

## 1. Introduction

Visual–spatial processing (Gv) is a broad ability in the Cattell–Horn–Carroll theory of cognitive abilities (Schneider and McGrew 2018; Smith 2004; Wu and Adams 2013). Across the literature, it is referred to as spatial reasoning, spatial thinking, spatial ability, or spatial visualization and is the ability to mentally visualize, rotate, transform, represent, and recognize symbolic information (Gilligan-Lee et al. 2022). While verbal and quantitative reasoning are widely assessed, spatial reasoning is another important aspect of an individual’s readiness to solve complex problems and excel in relevant professions (Cheng and Mix 2014; Wai et al. 2009).

Spatial reasoning skills have long been part of tests used for educational and occupational placement, such as the Armed Services Vocational Aptitude Battery (ASVAB; Defense Manpower Data Center 2006). Spatial reasoning is an important tool for problem solving in everyday life and in specific content areas, such as math and science (Cheng and Mix 2014; Wai and Lakin 2020; Wai et al. 2009; Young et al. 2018). An ability to interpret visual information to make inferences and to express one’s thinking visually is a critical tool in 21st-century careers (Gagnier and Fisher 2020; Newcombe 2010).

However, spatial tests have not seen the emphasis in formal educational settings that measures of verbal and quantitative reasoning have experienced, such as inclusion in widely used U.S. college admissions tests (SAT/ACT; Wai et al. 2009). The *Next Generation Science Standards* (Next Generation Science Standards 2013) include visualization skills in their Science & Engineering Practices. Therefore, measures of spatial reasoning may see a resurgence in educational use, alongside their continued relevance to research.

To support the expanded use of spatial tests in research and educational settings, newer psychometric models can help researchers to provide evidence for the validity of existing spatial reasoning item formats, including construct representation and response processes (Embretson 2002). These models may also support the development of new spatial test items and scales, particularly alongside automated item generation (AIG; Gierl et al. 2021).

In this study, we used an explanatory item response theory (IRT) approach to examine how various characteristics of spatial reasoning items influenced student responses and item characteristics. Explanatory IRT methods can test hypotheses related to underlying cognitive processes and support construct definitions. We specifically used the linear logistic test model (LLTM; Fischer 1973, 1997), which is an item-explanatory model that allows the researcher to specify a Q-matrix of item features that may reflect underlying cognitive processes that give rise to item responses. It is also used pragmatically to identify sources of item difficulty to guide item development.

### 1.1. Construct Representation, Response Processes, and Explanatory Item Response Theory

Cognitive psychology has long explored the component processes that underlie performance on ability test questions Zumbo and Hubley (2017). Some of this work is conducted with the goal of understanding the underlying brain processes that lead to intelligent behavior. These methods can also inform item construction and the accurate representation of the construct being measured (Embretson and Gorin 2001). Different item features have been found to increase item difficulty (as a proxy for complexity). Therefore, each item type (e.g., figural matrices, verbal analogies, etc.) requires a distinct model of item difficulty to inform item development (e.g., Daniel and Embretson 2010; Gorin and Embretson 2006).

Psychometric techniques such as multi-faceted latent trait models (Linacre 1989) and cognitive diagnostic models or diagnostic classification models Rupp et al. (2010) can provide insights into the impact of these sources of complexity to better understand spatial reasoning in general and to inform item revisions or development to more fully reflect the construct (e.g., Embretson and Gorin 2001; Embretson 2007).

### 1.2. Object Assembly and Its Item Characteristics

**Figure 1:**
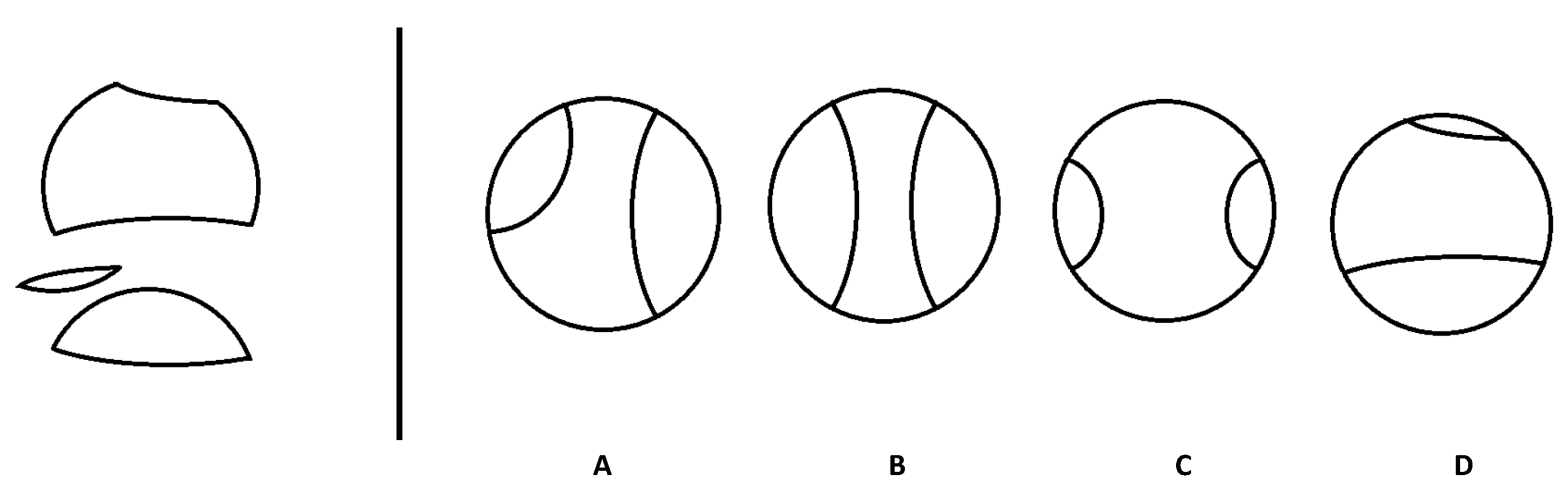
Example of an object assembly item. The pieces to the left of the line are the item stem, and the figures (**A**–**D**) are the options.

In this study, we focus on a spatial reasoning assessment format called *object assembly* (OA; also called the *Minnesota Paper Form Board Test* (MPFBT); Quasha and Likert 1937). OA is used in abilities research to measure specific visualization skills. It is also widely used in the Assembling Objects subtest on the ASVAB, which is used by the U.S. military for enlistment and placement in various training programs. OA items elicit an examinee’s visualization skills to determine which of the response options could be assembled from the pieces provided in the item question (stem); Figure 1 includes an example of this item format. A taxonomy of spatial tests (Eliot and Smith 1983) classifies OA as one example of a formboard task, which generally measures two-dimensional rotation and visualization skills (Lohman 1979).

Embretson and colleagues have studied the OA format for the assessment of spatial reasoning (Embretson and Gorin 2001; Ivie and Embretson 2010). For example, Embretson and Gorin (2001) built on cognitive process theory from Mumaw and Pellegrino (1984) and Pellegrino et al. (1985) and developed a multi-stage cognitive model for the OA format, which involves three stages: encoding, falsification, and confirmation. These stages and the associated processes were initially informed by Cooper and Shepard (1973)’s work on mental rotation, where they used the reaction time and systematically varied items to attempt to isolate the mental operations and representations that led to accurate responses. Pellegrino et al. (1985) extended this work to a format similar to object assembly, where they continued to isolate the processes and stages of problem solution.

Progressing this line of inquiry, Embretson and Gorin (2001) refined this model and categorized the variety of features into three phases, consistent with much of the work in cognitive process theory: encoding, falsification, and confirmation. The model implemented in Embretson and Gorin (2001) assumes the following cyclical process: first, test takers *encode* the entire item question (stem), including each piece, its shape and size, the number of edges, and the number of pieces; then, test takers use encoded stem pieces to analyze each distractor or alternative and *falsify* each incorrect answer; last, test takers process any remaining “non-falsifiable” distractor(s) and *confirm* that every stem piece matches the correct answer (key). Based on this model, Embretson and Gorin (2001) also identified nine item characteristics that influenced item difficulty. Specifically, the number of falsifiable distractors and the number of shapes with verbal labels were associated negatively with item difficulty, while the number of pieces, the total number of edges, the proportion of shapes mismatched by angular disparities, the number of comparison cycles, the number of displaced pieces, and the number of rotated pieces were associated positively with item difficulty. With these characteristics in mind, researchers can directly examine how spatial reasoning, as measured by a specific item form, is represented in a set of existing items as evidence of construct-related validity, and also use the components to systematically assemble items to represent the construct based on this theory.

In a related study, Ivie and Embretson (2010) investigated the cognitive process in solving the OA items on the MPFBT and refined their three-stage cognitive processing model. Their hierarchical regression and linear logistic test model (LLTM) results indicated that a set of updated item characteristics significantly contributed to item difficulty. In the encoding stage, characteristics included (1) the number of total pieces in the item stem; (2) the maximum number of edges on any one piece in the stem; (3) the number of pieces in the stem with verbal labels (e.g., circle, football, pie piece, etc.). In the falsification stage, characteristics included (4) the expected number of distractors falsifiable by each piece; (5) the expected number of cycles necessary to falsify the non-falsifiable distractors (NFDs, alternatives that cannot be falsified at first glance). Finally, in the confirmation stage, characteristics included (6) the number of displaced pieces (pieces that must be moved from their position in the stem to their position in the key) and (7) the number of pieces that must be rotated to match the stem to the key.

Concerning their impact on item difficulty, Ivie and Embretson (2010) found that the number of pieces, the expected number of distractors falsified, and the displacement between the stem and key had a significant negative effect on item difficulty, whereas the number of pieces with verbal labels, the more difficult falsification of NFD, and the necessary mental rotation had a significant positive effect on item difficulty. However, because they used existing measures (specifically the MPFBT) that were not designed to span these specific characteristics, their findings were limited by a lack of variability in the item features expected to contribute to complexity and thereby correlate with item difficulty.

Prior research demonstrates the prospective utility of item characteristics in engendering items of a specific level of difficulty through the discerning selection of pertinent attributes. In this study, we developed new items informed by previous studies aligned with the model of item difficulty proposed by Ivie and Embretson (2010). We were also interested in measuring spatial reasoning skills for younger students (ages 7–12), whereas previous work has focused on adult samples. Therefore, this project was a replication and extension of this area of research to more variable items and a younger population. Our work also has broader implications for the exploration and validation of item process theories.

### 1.3. The Current Study

This study aimed to explore the influence of item characteristics that contributed to differences in student performance related to spatial reasoning in the OA format. The following research questions guided our analysis:

1. What are the overall psychometric characteristics of an object assembly task used to assess spatial ability?
2. How do the characteristics of the object assembly items contribute to item difficulty?

### 1.4. Linear Logistic Test Model

The guiding framework for this study was explanatory item response theory (de Boeck and Wilson 2004). Explanatory IRT allows researchers to examine the impact of researcher-specified item characteristics and person characteristics on item responses. In the context of spatial reasoning assessment, explanatory IRT can help researchers to explore the impact of various spatial characteristics on item difficulty. Specifically, item-explanatory models such as the linear logistic test model (LLTM; Fischer 1973, 1997) are well suited to this context.

The LLTM models item properties such as item difficulty using researcher-specified item characteristics that are expected to contribute to differences in item difficulty. Item characteristics are specified using a Q-matrix, a binary matrix, that classifies each item according to characteristics that are expected to relate to item difficulty. In this Q-matrix, the rows stand for items and the columns represents item characteristics. Each element in the Q-matrix identifies whether a certain item characteristic is featured by a specific item. If the characteristic is featured, the element takes the value of 1; otherwise, it is marked as 0. After the Q-matrix is specified, the influence of the item characteristics on item difficulty is estimated alongside overall item difficulty and examinee ability parameters. Although various adaptations of the LLTM have been proposed (Embretson 2016), the traditional formulation of the LLTM is an extension of the dichotomous Rasch model (Rasch 1960) that can be represented as follows. First, the dichotomous Rasch model is specified as

(1) $$P\left(X_{ni}=1\right)=\frac{e^{\theta_{n}-\delta_{i}}}{1+e^{\theta_{n}-\delta_{i}}},$$

where $P(X_{ni}=1)$ is the probability that examinee *n* provides a correct response (X=1) on item *i*, $\theta_{n}$ is the estimated person location parameter (i.e., ability) for examinee *n*, and $\delta_{i}$ is the predicted item location parameter (i.e., difficulty) based on scored item characteristics. In the LLTM, the predicted item location $\delta_{i}^{\prime }$ and person location $\theta_{n}^{\prime }$ are estimated in the same way as in the Rasch model. Additionally, $\delta_{i}^{\prime }$ reflects the impact of item characteristics, which is specified as

(2) $$\delta_{i}^{\prime }=\sum_{m=1}^{M}\eta_{m}q_{im}+\eta_{0},$$

where $q_{im}$ is the score on item characteristic *m* (m=1,2,…,M) for item *i*, $\eta_{m}$ is the estimated weight of item characteristic *m*, and $\eta_{0}$ is a normalization constant. When the LLTM is applied, item characteristic weights are directly estimated, and these estimates are used to calculate $\delta_{i}^{\prime }$.

Researchers can use the LLTM to empirically examine the contribution of item characteristics to item difficulty on the same scale as item and examinee parameters. Researchers can use the direction and magnitude of the item characteristic parameter estimates to inform the interpretation and use of assessment results, inform their understanding of the construct, identify areas for revision, and inform the development of new items targeted toward specific characteristics or combinations of characteristics.

Researchers have used LLTM methods in limited cases to examine the contributions of spatial characteristics to item difficulty in spatial reasoning assessments (e.g., Embretson and Gorin 2001; Ivie and Embretson 2010). In this study, we applied an LLTM to examine the generalizability of this model to new items and a younger population. This work also provides a model for the use of LLTM to explore cognitive processes with other constructs and item formats.

## 2. Materials and Methods

### 2.1. Participants

This study was conducted as part of a larger project whose goal was to develop a spatial reasoning battery for students in grades 2–8. To ensure that test items had sufficient variability in difficulty, a new form of 15 items was developed as part of this work. We collected two focal samples for this study to increase respondent variability on the latent trait. First, we recruited 73 students in grades 3 to 7 through summer camp programs that were focused on a range of academic, artistic, and STEM-related topics. Students in this sample were aged 9–12 (median = 11), with 44% female, 65% white, 21% Asian, 7% Black, and 7% other. These camps occurred at either a southeastern university or a midwestern university. Parents or guardians of students were approached to have their students participate in the research study. Students received a giftcard for their participation. Second, we collected data from 101 undergraduate students from a southeastern university, primarily Education majors, to augment our sample. These respondents were 68% female, 81% white, 10% Black, 7% Asian, and 3% other groups. We did not collect their ages. Results from preliminary analyses indicated that these two samples could be combined for analyses (discussed further in the Results section). These students were recruited through their college’s research portal and offered extra credit in their coursework for their participation. All data were collected under the approval of the Institutional Review Board of the university.

### 2.2. Instrument

An initial set of object assembly items was developed relying on older versions of the test format and prior work on their cognitive processes. Based on pilot testing, fifteen items with good classical discrimination statistics and a range of difficulty were selected for this study. Our list of item characteristics was based on characteristics defined by Embretson and Gorin (2001) that showed potential in their original analyses to explain item difficulty (see also Ivie and Embretson 2010). Some of the item characteristics were self-explanatory while others were less so. *Number of pieces* (Npieces), *total edges* (Tedges) across pieces, *maximum edges* (Medges) on any one piece, and *curved pieces* (Cpieces) were judged by the test developer and checked by a collaborator for accuracy. These characteristics described the stem.

The item developer and a collaborator rated the more subjective item characteristics concurrently. For example, the decision of whether a distractor was “easily excluded” (EED) could vary by rater. Using the definitions provided in Table 1, two raters independently categorized each item. The few discrepancies were discussed and resolved. *Pieces with labels* (Lpieces) was a measure of how many pieces in the stem had clear labels (square, triangle, [pie] slice). Irregular shapes without obvious labels were not counted. *Regular-shape solution* (RSS) was judged based on the key having a standard shape (circle, equilateral triangle, right triangle, or square). *Displaced pieces* (Dpieces) was based on the number of pieces in the stem that were moved to a different location in the key. *Rotated pieces* (Rpieces) was based on the pieces in the key that had to be rotated from the key to the stem to reach the correct answer. *Easily excluded distractors* (EED; called falsifiable distractors by Embretson and Gorin 2001) was the most subjective characteristic and included any distractors with a different number of pieces or obviously different shapes from the stem. A description of the item characteristics is presented in Table 1.

Figure 1 shows an example item. In this item, *Npieces* is 3; *Tedges* is 8; *Medges* is 4; *Cpieces* is 3; *Lpieces* is 0; *RSS* is 1 (yes; the key has a circle shape); *Dpieces* is 2; *Rpieces* is 2; and *EED* is 0 (all options have the same number of pieces as the stem).

### 2.3. Data Analysis

We analyzed the OA items using three steps. First, we evaluated the instrument’s overall psychometric characteristics. Specifically, we evaluated the internal consistency using Cronbach’s alpha, which assesses the degree of item covariance on a 0 to 1 scale. An α value closer to 1 indicates a stronger correlation among the items, implying that there are consistent response patterns between items. Additionally, we calculated descriptive statistics for scored item responses for each item, including the mean (or the proportion of correct responses), standard deviation, and corrected item–total correlation, which is the correlation for the item with the total scores without this item. This analysis gave us preliminary insights into the degree to which the OA items could be interpreted as a measure of spatial reasoning. We used the *psych* (Procedures for Psychological, Psychometric, and Personality Research; Revelle 2023) package to conduct these analyses in *R* R Core Team (2022).

Then, we analyzed the responses using the dichotomous Rasch model (Rasch 1960) via the *eRm* (Mair et al. 2021) *R* package. We selected this model for several reasons. First, Rasch models are well suited to relatively small sample sizes compared to the requirements for other, more complex, parametric IRT models. For example, researchers have noted that it is possible to obtain stable estimates with the dichotomous Rasch model with samples as small as n = 30 participants (Bond et al. 2020; Linacre 1994). Second, this model allowed us to evaluate the characteristics of the OA items before we explored the contributions of item characteristics to item difficulty. As Green and Smith (1987) pointed out, evidence of adequate psychometric characteristics, including acceptable item fit, is essential before the results of extended IRT models can be meaningfully interpreted. Accordingly, we evaluated item properties based on the dichotomous Rasch model as a preliminary step in our LLTM analysis. This model allowed us to explore the degree to which the OA items reflected a unidimensional construct in which items exhibited useful psychometric characteristics. Specifically, unidimensionality was examined with a principal components analysis of standardized residuals. A maximum eigenvalue of 2.00 is recommended for sufficient unidimensionality (Chou and Wang 2010). We evaluated the overall model fit using a likelihood ratio test.

Additionally, the Rasch model assumes that the items exhibit local independence—such that, after controlling for the primary latent variable, item responses are statistically independent. We evaluated this assumption by calculating the residual correlations between each pair of items after controlling for the model. The absolute value of the correlation coefficients is recommended to be less than 0.2 to indicate adherence to local independence (Linacre 1994). Furthermore, the infit and outfit mean square error (MSE) statistics for items and respondents assist in uncovering the adherence to invariant item difficulty across participants (i.e., item difficulty is the same for all participants) and invariant person locations across items (i.e., person estimates of spatial ability do not depend on the specific items). Specifically, a value of 1.0 for both infit and outfit *MSE* and a value of 0.0 for both infit and outfit *z* indicate good fit.

To understand the ability of our items in discriminating the two focal samples (subgroups), a differential item functioning (DIF; Wright and Masters 1982) analysis was conducted. Specifically, using a concurrent calibration approach, we estimated the item difficulty and standard errors specific to each subgroup with the dichotomous Rasch model and calculated the standardized differences in item difficulty between the two subgroups given by

(3) $$z=\frac{d_{1}-d_{2}}{\sqrt{se_{1}^{2}+se_{2}^{2}}},$$

where *z* is the standardized difference, $d_{1}$ and $d_{2}$ are the item difficulty specific to subgroups 1 and 2, respectively, and $se_{1}$ and $se_{2}$ are the standard errors of the item difficulty specific to subgroups 1 and 2, respectively. Higher values of *z* indicate greater item locations (more difficult) for subgroup 1 compared to subgroup 2.

Finally, we applied the LLTM to the scored OA responses using the eRm (Mair et al. 2021) package with the Q-matrix illustrated in Table 2. Item classifications shown in the Q-matrix were specified based on expert classification of the OA items related to nine of the characteristics included by (Embretson and Gorin 2001; see also Ivie and Embretson 2010). To facilitate interpretability and model robustness, we dichotomized the polytomous characteristics. For each of these characteristics, we calculated the mean of all the unique values and coded the values lower than the mean as “0” and the values higher than or equal to the mean as “1” (i.e., a mean split). For instance, the *Npieces* for the 15 items were [2, 2, 2, 3, 5, 4, 4, 3, 5, 3, 4, 4, 4, 5, 4], and the unique values were 2, 3, 4, and 5, with a mean of 3.5. The values lower than 3.5 (2 and 3) were denoted as 0, and the values equal to or above 3.5 (4 and 5) were denoted as 1. As a result, the dichotomized *Npieces* became [0, 0, 0, 0, 1, 1, 1, 0, 1, 0, 1, 1, 1, 1, 1]. This resulted in acceptable variability in each dichotomized variable.

We evaluated the fit of the LLTM in three ways, following Baghaei and Kubinger (2015). First, we used the log-likelihood chi-square test for both the dichotomous Rasch model and the LLTM, and we compared the difference between the −2log-likelihoods of the two models against a critical value of chi-square (i.e., the value at the 0.95 quantile, α=0.05) with the degrees of freedom equal to the difference between the number of parameters in the two models Fischer (1973). A difference between the −2log-likelihoods less than the corresponding critical value indicated a good fit for the LLTM, implying that the identified item characteristics appreciably accounted for the item difficulty parameters. Second, we calculated Pearson’s correlation coefficients between the item difficulty parameters (δ) of the dichotomous Rasch model and the item difficulty parameters ($\delta^{\prime }$) based on the LLTM. The coefficient takes a value between 0 and 1, and higher values indicated that the item characteristics in the LLTM accounted for more variance in the item difficulty estimated by the dichotomous Rasch model. Third, we examined the alignment between the item difficulty parameters (i.e., $\delta^{\prime }$ and δ) of the LLTM and the dichotomous Rasch model. To do this, we normalized and plotted the LLTM estimates against the item difficulty parameters of the dichotomous Rasch model.

After we examined these fit indices, we evaluated the LLTM results to better understand the influence of specific item characteristics on item difficulty. We examined the $\delta^{\prime }$ parameter for each item and the η parameter for each item characteristic with their standard errors and 95% confidence intervals. Both the $\delta^{\prime }$ and η parameters were estimated on a log-odds (i.e., “logit”) scale. The $\delta^{\prime }$ parameter in the LLTM was interpreted in the same way as the *b* or difficulty parameter in the dichotomous Rasch model. Specifically, a larger value of the $\delta^{\prime }$ parameter indicated that the corresponding item was more difficult. A larger value of the η parameter indicated that the corresponding item characteristic made the items more difficult.

## 3. Results

Preliminary analyses indicated that the OA items exhibited acceptable internal consistency (standardized α=0.88). Table 3 presents summary statistics for the OA items. Generally, the items demonstrated moderate difficulty and positive correlations with the total score. The item with the highest proportion correct was Item 1 (M=0.87), and Items 8 and 15 had the lowest proportion correct (M=0.43); these items demonstrated moderate difficulty for our combined sample. The item with the highest corrected item–total correlation was Item 5 (r=0.71), and the item with the lowest corrected item–total correlation was Item 11 (r=0.35). The moderate, positive values for the corrected item–total correlation statistics corresponded to our finding of adequate internal consistency for the OA items in our sample. This warranted a further analysis of these items.

### 3.1. Dichotomous Rasch Model

Results from the dichotomous Rasch model analysis suggested that the OA item responses generally adhered to the model requirements, with the likelihood ratio test of model fit yielding $\chi^{2}\left(11\right)=16.79$, p=0.114. The Rasch model estimates for items and persons explained 44.09% of the variance in the scored responses, indicating a large effect size (Cohen 1992). In addition, a principal components analysis of the standardized residuals from the model (Chou and Wang 2010) indicated eigenvalues for the model contrasts less than or equal to 1.94, which was lower than the recommended maximum value of 2.00 for sufficient unidimensionality for Rasch model analyses (Chou and Wang 2010); this result suggested that, after controlling for the primary latent variable, there were no meaningful secondary dimensions in the data. In addition, correlations between the residuals associated with individual items were low (M=0.06)—indicating adherence to local independence. Finally, average values for individual item fit statistics (infit *MSE*: M=0.95, SD=0.21; outfit *MSE*: M=0.90, SD=0.35) and person fit statistics (infit *MSE*: M=0.98, SD=0.26; outfit *MSE*: M=0.90, SD=0.64) were within the generally expected ranges when the data fit the Rasch model (Smith 2004; Wu and Adams 2013).

Table 4 provides the item calibrations for the 15 items, along with their difficulty parameters, standard errors, 95% confidence intervals, and item fit estimates from the dichotomous Rasch model. The model was specified such that higher item location estimates corresponded to lower proportions of correct responses (i.e., more difficult items). The reliability of item separation was equal to 0.99, suggesting differences in the level of latent ability required to correctly respond to the OA items. Overall item difficulty estimates ranged from −1.92 logits (SE=0.29) for Item 1, which was the easiest item, to 1.79 logits (SE=0.23) for Item 15, which was the most difficult item. These results suggest that there were differences in the level of spatial ability required to correctly respond to the OA items.

Examination of the item fit statistics indicated an overall adequate fit to the dichotomous Rasch model. Specifically, the highest values of fit statistics were observed for Item 11 (outfit MSE=1.32; outfit z=1.20; infit MSE=1.36; infit z=2.70), and the lowest values of fit statistics were observed for Item 2 (outfit MSE=0.35; outfit z=−1.77; infit MSE=0.65; infit z=−2.80) and Item 1 (outfit MSE=0.36; outfit z=−1.28; infit MSE=0.73; infit z=−1.70). Despite this variation, these item-level fit statistics were within the ranges that are generally considered acceptable for Rasch model analyses (Engelhard and Wang 2021). Overall, the results of the dichotomous Rasch model analysis supported the use of the LLTM to explore item difficulty in more detail using item characteristics.

#### DIF Analysis of the Two Subgroups

**Figure 2:**
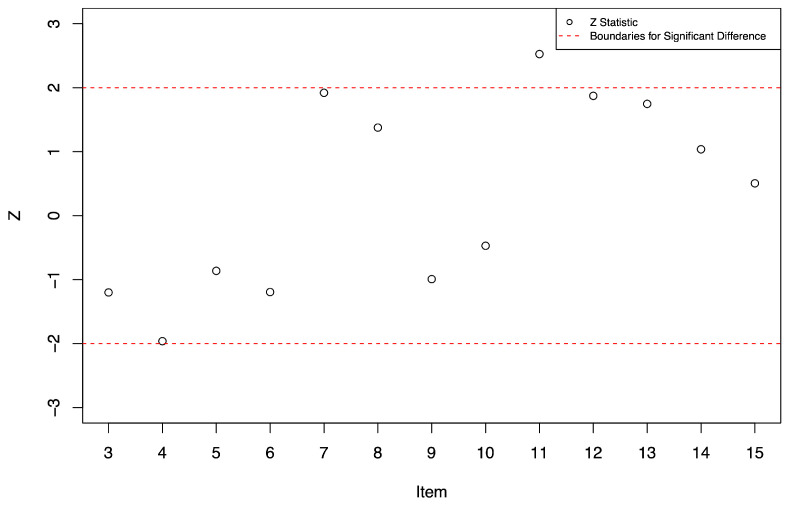
Plot of standardized differences (*z*) in subgroup-specific item difficulty estimates between subgroups.

Due to some constant response patterns within subgroups (all items were either correct or incorrect within one or both groups), Items 1 and 2 were excluded from the DIF analysis. Table 5 presents the item difficulty estimates and standard errors specific to each subgroup, *z* statistics, and *p* values. Subgroup 1 represents the focal group of children and subgroup 2 the group of undergraduate students. According to the results, the most difficult item for subgroup 1 compared to subgroup 2 was Item 11 (*z* = 2.53, *p* < .05), followed by Item 7 (*z* = 1.92, *p* = .05), and the easiest item for subgroup 1 compared to subgroup 2 was Item 4 (*z* = −1.96, *p* = .05). The remaining items were not significantly different in their difficulty between subgroups. Nevertheless, there were both positive and negative z statistics, indicating that some items were easier for subgroup 1 whereas others were easier for subgroup 2. The z statistics for Items 3 to 15 are also presented in a plot in Figure 2. Specifically, the x-axis presents the items and the y-axis the z statistics. Dashed horizontal lines at +2 and −2 demarcate statistically significant deviance in item difficulty between subgroups.

### 3.2. LLTM

**Figure 3:**
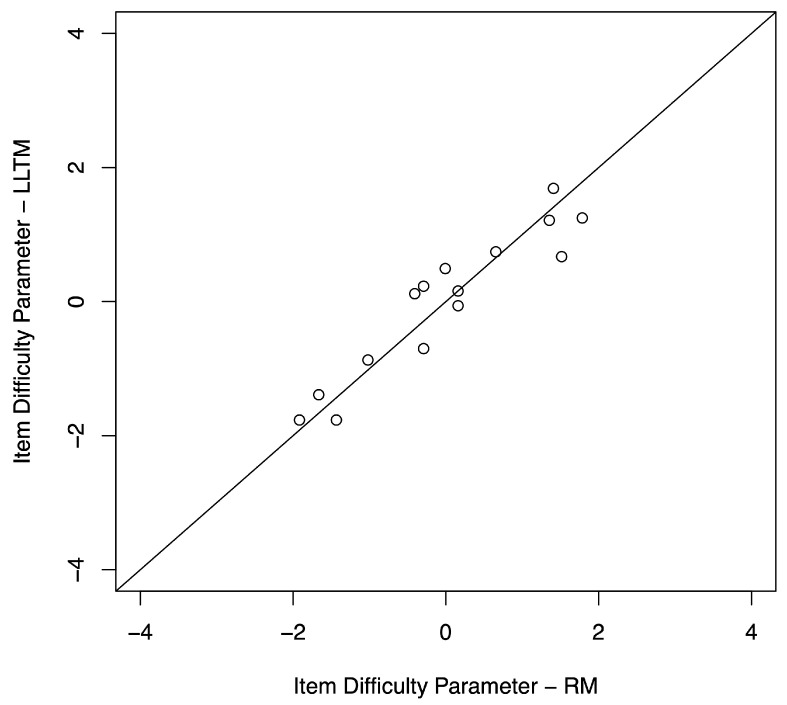
Scatterplot of RM item parameters against LLTM item parameters.

The model fit analyses for the LLTM yielded somewhat divergent results. Specifically, the −2log-likelihood statistics were equal to 1137.751 for the dichotomous Rasch model and 1191.925 for the LLTM, with a difference in −2log-likelihoods of 54.174. The difference in the degrees of freedom between the two models was 5. The 0.95 quantile (α=0.05) of the $\chi^{2}$ distribution with df=5 was 11.0705. The −2log-likelihood difference was greater than 11.0705, indicating that the dichotomous Rasch model had a better fit than the LLTM, and the specified item characteristics in the LLTM did not capture all variation in item difficulty in the dichotomous Rasch model. This result was consistent with previous studies that have used the likelihood ratio test to compare the LLTM and standard Rasch models (Alexandrowicz 2011; Baghaei and Hohensinn 2017; Baghaei and Ravand 2015; Cao et al. 2014; Fischer 2005; Fischer and Formann 1982), even when the Q-matrix was well constructed and item characteristics accounted for most of the variance in item difficulty Rijmen and de Boeck (2002). The LLTM assumes that the variance of item difficulty is completely accounted for by item characteristics (Cao et al. 2014), which may explain why the likelihood ratio test result is always significant. Nevertheless, despite the rejection of the likelihood ratio test between the Rasch model and the LLTM, the correlation coefficient between the difficulty parameters of the two models was strong and positive (r=0.94), indicating that approximately 88.36% of the variance in overall item difficulty could be accounted for by the nine spatial characteristics. Illustrating this correspondence, Figure 3 shows a scatterplot of the LLTM item difficulty parameters and the item difficulty parameters from the dichotomous Rasch model. Item estimates are generally located along the diagonal, indicating concurrence between the models. The alignment in the item location estimates between these two models reflects the degree to which the item characteristics in the LLTM accurately reflected the difficulty of the items. In other words, the correspondence between item estimates from the Rasch model and the LLTM suggests that the spatial characteristics from the LLTM meaningfully contributed to item difficulty. Together, these results suggest that the item characteristics provide useful information about differences in item difficulty in the context of the OA assessment.

Table 6 summarizes results from the LLTM analysis related to the nine item characteristics. Some of the features associated with greater difficulty (complexity) included *Dpieces*, which contributed the most to difficulty (η=1.89), possibly because it required the most visual encoding of individual shapes to find the correct answer. *EED* was the easiest characteristic (η=−1.19), meaning that including obviously incorrect distractors made items much easier. These findings were consistent with the findings of previous studies (Embretson and Gorin 2001; Ivie and Embretson 2010). The 95% confidence interval reported for each eta parameter showed that all characteristics except RSS were statistically significant at p<.05, which indicated that these characteristics substantially contributed to the item difficulty. Based on the absolute value of eta parameters, the smallest effect estimate that did not include zero in the confidence interval was *Rpieces* (|η|=0.44), implying that rotating a large number of pieces in the key from the stem did not make an item more difficult in practice, which was contradictory to Ivie and Embretson’s (2010) finding that more mental rotation necessarily resulted in more difficult items. Differences in the populations or items sampled may explain our divergent findings.

**Figure 4:**
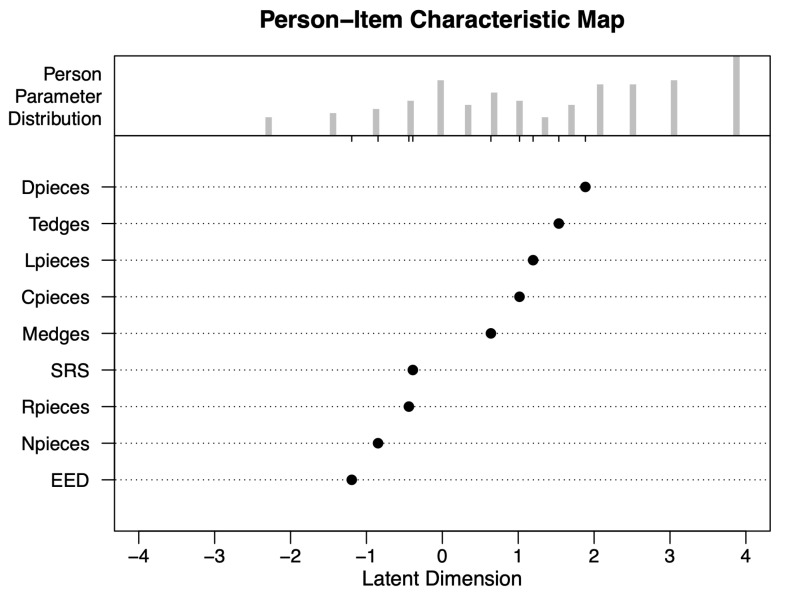
The personp–item characteristic Wright map of the LLTM.

Figure 4 illustrates the calibrations of the item characteristics and respondents on the logit scale that represents the latent construct, OA. The calibrations shown in this figure correspond to the calibrations presented in Table 6 for the nine item characteristics. Respondent locations on the latent construct are illustrated using the frequency histogram at the top. The x-axis at the bottom (latent dimension) shows the logit scale. Higher numbers correspond to higher OA ability for respondents and more difficult item characteristics, and lower numbers correspond to lower OA ability for respondents and easier item characteristics. Item characteristic locations on the latent construct are plotted, with each characteristic marked on the y-axis on the left. A close examination of the respondent estimates indicates that the respondents were not normally distributed, as expected from the fact that we collected two sample groups with different OA abilities. The middle of the respondent ability on the logit scale is approximately θ=1.40, while the item characteristic locations center around η=0.2. This indicates that the item characteristics were relatively easy for this group of participants. However, considering the respondent with the lowest OA ability being around θ=−2.0, the spread of respondent locations being rather even, and the items targeting students in grades 2–8, the item characteristics were well targeted to our sample.

In summary, Figure 4 presents the respondents and item characteristics distributed on a scale that reflects their ability (for respondents) and difficulty (for item characteristics). The histogram at the top displays the frequency of respondents falling within certain ability levels. On the left, each characteristic is plotted to show where it lies in terms of difficulty. Despite the wide range of participant abilities, the item characteristics seem to be suitably challenging for our target population.

Table 7 shows the LLTM item difficulty parameters, based on the item characteristic parameters. Since the LLTM assumes a linear combination of item characteristics as item difficulty, the beta estimates in this table are the sum of the eta values of certain item characteristics that are involved in a particular item. Accordingly, considering the effect of the item characteristics, the most difficult item was Item 14 ($\delta^{\prime }=-2.51$) and the easiest items were Items 1 and 2 ($\delta^{\prime }=-0.94$).

## 4. Discussion

We used explanatory IRT to examine the impact of various spatial characteristics on the difficulty of OA items in a spatial reasoning assessment. This approach allowed us to empirically test the degree to which certain spatial characteristics impacted examinee performance. Our approach reflects previous work in which researchers (e.g., Embretson and Gorin 2001; Ivie and Embretson 2010) have used explanatory IRT models to understand the contributions of spatial characteristics to examinee performance as a method to improve theories related to spatial reasoning and improve item development procedures for future assessment procedures. In this section, we discuss our results as they relate to our guiding research questions. Then, we discuss the implications of our work for research and practice.

### 4.1. What Are the Overall Psychometric Characteristics of Object Assembly?

Our first research question asked about the overall psychometric properties of the OA assessment. We addressed this research question by examining classical psychometric indicators of item difficulty and discrimination, and by examining item calibrations and fit statistics from the dichotomous Rasch model. Specifically, we observed acceptable levels of internal consistency and variation in student responses to each item. These results suggest that the OA items were generally internally consistent and discriminated well among students with different levels of spatial reasoning skills. Overall, we found that the OA items exhibited acceptable psychometric properties that supported more detailed explorations of student responses.

### 4.2. How Do the Characteristics of the Object Assembly Items Contribute to Item Difficulty?

Given the generally acceptable psychometric properties for the OA items, we proceeded with an explanatory IRT analysis to examine the contributions of various spatial reasoning skills to item difficulty in detail. We used a dichotomous specification of the LLTM to examine the degree to which our researcher-specified Q-matrix of item characteristics could explain student responses to the OA items. This analysis was essentially a test of our theory about how the spatial characteristics represented in our items may have contributed to differences in student responses. Our results suggested that the specified item characteristics were effective in predicting item difficulty. In other words, we identified components of spatial reasoning that contributed to student performance on OA items. Specifically, we found that whether the key has a standard shape does not significantly contribute to item difficulty, but item difficulty is significantly affected by the number of displaced pieces in the key, rather than the number of pieces that are rotated.

### 4.3. Implications

Assessing specific spatial reasoning abilities is critical for certain specialties and jobs that require these skills. It is paramount to develop measurement instruments that effectively measure spatial ability to support the research and practice related to this construct.

With regard to assessing spatial reasoning, our study has implications for the development and interpretation of OA tasks specifically. Our findings indicate that the characteristics underlying item difficulty are consistent for new items and a broader sample of participant ages.

Reflecting previous work in which researchers have used explanatory IRT models in spatial reasoning assessments, we demonstrated how the LLTM could be applied to evaluate a set of spatial reasoning items, providing evidence that variations in spatial characteristics contributed in expected ways to variations in student performance in a younger population than has previously been considered. This approach provides a source of construct-related validity evidence that can be used to support the interpretation and use of OA items as a measure of spatial reasoning.

More generally, our research has implications for assessment development related to spatial reasoning. In previous studies (Embretson and Gorin 2001; Ivie and Embretson 2010), researchers have used explanatory IRT methods to demonstrate how estimates of item characteristics can inform item development and test assembly for spatial reasoning using test-taker populations composed of college-aged students. Our work demonstrates the use of this approach with younger populations as a means of exploring the nature of spatial reasoning and informing theory-driven item development for this population. For example, researchers and practitioners could use the results from explanatory IRT analyses, such as the LLTM approach illustrated in this study, to identify characteristics that contribute to item difficulty for spatial reasoning items, and then use these characteristics to develop items that are targeted to various components of spatial reasoning at varying levels of difficulty. Likewise, researchers could use this approach to test theories about how other aspects of spatial reasoning contribute to item difficulty for OA items or other spatial item types.

### 4.4. Limitations

Our study has several limitations that warrant further research. First, our analyses were limited to a single OA assessment administered. In future studies, researchers could apply the methods that we demonstrate here with other spatial reasoning assessments, including other item types, as well as with samples with different characteristics. Additionally, we did not consider how the influence of spatial characteristics on item difficulty may have varied across students with different characteristics (e.g., demographic subgroups). In future studies, researchers could consider the degree to which item characteristics may function differently across student subgroups.

### 4.5. Directions for Future Research

The current study focused on the post-hoc analysis of item characteristics and their relationships with item difficulty. Future research should look at the predictive value of these characteristics in either constructing or predicting the difficulty of new items. Our next step in this line of research is to apply the Q-matrix to a new set of items to estimate the shrinkage of the model on a validation data set. If these item characteristics predict item difficulty efficiently on a validation pool of items, we will develop new pools of items based on these dimensions. Complementing this, it would also be valuable for future research to administer both the MPFBT and these new OA items concurrently. This combined administration could offer important insights into the relative difficulty of these items, further enhancing our understanding of item characteristics in relation to their difficulty.

In addition, explanatory IRT in general, including models such as the LLTM, may prove useful in many ways to automated item generation (AIG) procedures. Both AIG item models and the Q-matrices of explanatory IRT models are dependent on subject matter experts to define the key features that contribute to construct validity and/or item difficulty. Thus, item-explanatory models such as the LLTM could be used to validate item models created by subject matter experts (as Loe et al. 2018 did) or to guide the creation of item models in the future. Future research is needed to explore the degree to which the characteristics that we identified as significant predictors function similarly with different items.

Finally, it is important to note that identifying predictors of item difficulty does not necessarily ensure construct representation. This work was one piece of a larger study to understand the assessment of spatial reasoning in students in late elementary through middle school. In future studies, researchers can use techniques such as the item component modeling approach illustrated here to explore the construct of spatial reasoning in more detail.

## Figures and Tables

**Table 1:** Descriptions of item characteristics.

Item Characteristics	Description
Number of pieces (Npieces)	The number of pieces in the stem
Total edges (Tedges)	The total number of edges across pieces in the stem
Maximum edges (Medges)	The maximum number of edges on any one piece in the stem
Curved pieces (Cpieces)	Pieces in the stem containing at least one curved edge
Pieces with labels (Lpieces)	All pieces in the stem with clear labels (square, triangle, [pie] slice)
Regular-shape solution (RSS)	The key has a standard shape (circle, equilateral triangle, right triangle, or square)
Displaced pieces (Dpieces)	The number of pieces in the stem that were moved to a different location in the key
Rotated pieces (Rpieces)	The number of pieces in the key that had to be rotated from the key to the stem to reach the correct answer
Easily excluded distractors (EED)	The number of distractors with a different number of pieces or obviously different shapes from the stem

**Table 2:** Q-matrix for the 15 OA items.

Item	Npieces	Lpieces	Tedges	Medges	Cpieces	EED	RSS	Dpieces	Rpieces
1	0	0	0	1	0	1	1	0	0
2	0	0	0	1	0	1	1	0	0
3	0	0	0	0	1	1	1	0	0
4	0	0	1	0	0	1	1	0	0
5	1	0	1	0	1	0	1	0	0
6	1	1	1	1	0	1	1	0	0
7	1	0	1	1	1	1	1	0	0
8	0	0	1	1	0	1	1	1	1
9	1	0	1	0	1	1	1	0	0
10	0	0	1	1	0	1	0	0	0
11	1	1	1	0	0	0	1	0	1
12	1	1	1	0	0	0	1	0	0
13	1	0	1	0	1	1	1	1	1
14	1	1	1	0	1	0	1	0	0
15	1	1	1	0	1	0	1	0	1

Notes. For characteristics 1, 3, 5, 8, and 9, a value of 1 indicates that an item was higher than or equal to the mean on a certain characteristic, and a value of 0 indicates that an item was lower than the mean on a certain characteristic. All others are yes/no. Npieces = number of pieces; Lpieces = pieces with labels; Tegdes = total edges; Medges = maximum edges; Cpieces = curved pieces; EED = easily excluded distractors; RSS = regular-shape solution; Dpieces = displaced pieces; Rpieces = rotated pieces.

**Table 3:** Item statistics.

Item	n	Proportion Correct (Mean)	SD	Corrected Item–Total Correlation
1	123	0.87	0.34	0.51
2	123	0.82	0.39	0.60
3	123	0.85	0.36	0.48
4	123	0.77	0.42	0.59
5	123	0.63	0.48	0.71
6	123	0.69	0.46	0.58
7	123	0.61	0.49	0.49
8	123	0.43	0.50	0.49
9	170	0.68	0.47	0.67
10	170	0.65	0.48	0.52
11	169	0.64	0.48	0.35
12	170	0.46	0.50	0.53
13	169	0.52	0.50	0.45
14	168	0.50	0.50	0.53
15	170	0.43	0.50	0.54

Note. SD = standard deviation; corrected item–total correlation = correlation for this item with total scores without this item.

**Table 4:** Item calibrations from the dichotomous Rasch model.

Item	δ	*SE*	Lower CI	Upper CI	Outfit *MSE*	Infit *MSE*	Outfit *z*	Infit *z*
1	−1.92	0.29	−1.35	−2.49	0.36	0.73	−1.28	−1.70
2	−1.43	0.27	−0.92	−1.95	0.35	0.65	−1.77	−2.80 *
3	−1.66	0.28	−1.12	−2.21	0.85	0.81	−0.15	−1.24
4	−1.02	0.25	−0.53	−1.51	0.52	0.79	−1.46	−1.70
5	−0.01	0.23	0.44	−0.46	0.52	0.70	−2.44	−2.70
6	−0.41	0.24	0.05	−0.87	0.77	0.90	−0.83	−0.82
7	0.16	0.23	0.61	−0.29	1.26	1.15	1.16	1.16
8	1.35	0.23	1.81	0.90	1.21	1.10	0.86	0.80
9	−0.29	0.23	0.17	−0.75	0.62	0.79	−1.62	−1.85
10	0.16	0.23	0.61	−0.29	1.18	1.15	0.85	1.16
11	−0.29	0.23	0.17	−0.75	1.32	1.36	1.20	2.70
12	1.52	0.23	1.97	1.06	1.13	1.01	0.56	0.11
13	0.65	0.23	1.10	0.21	1.26	1.20	1.22	1.51
14	1.41	0.23	1.86	0.96	1.15	1.05	0.62	0.44
15	1.79	0.23	2.24	1.33	1.04	1.01	0.22	0.16
*M*	0.00	0.24	0.47	−0.47	0.90	0.96	−0.19	−0.32
*SD*	1.19	0.02	1.15	1.22	0.35	0.21	1.26	1.65

Note. *SE* = standard error; CI = confidence interval; the upper and lower interval range is for 95% confidence; *MSE* = mean square error; * *p* < .05.

**Table 5:** Results for DIF analysis across subgroups.

Item	$d_{1}$	${\mathrm{se}}_{1}$	$d_{2}$	${\mathrm{se}}_{2}$	*z*	*p*
3	−2.81	0.96	−1.61	0.29	−1.20	0.23
4	−2.81	0.96	−0.86	0.28	−1.96	0.05 *
5	−0.43	0.40	0.01	0.30	−0.86	0.39
6	−1.03	0.48	−0.36	0.29	−1.19	0.23
7	0.49	0.33	−0.36	0.29	1.92	0.05 *
8	1.46	0.30	0.82	0.36	1.38	0.17
9	−0.80	0.45	−0.27	0.29	−0.99	0.32
10	−0.12	0.37	0.11	0.31	−0.47	0.64
11	0.26	0.35	−0.86	0.28	2.53	0.01 *
12	1.69	0.30	0.82	0.36	1.87	0.06
13	0.88	0.31	0.11	0.31	1.75	0.08
14	1.46	0.30	0.97	0.37	1.04	0.30
15	1.76	0.30	1.50	0.44	0.51	0.61

Note. $d_{1}=$ item difficulty estimates specific to subgroup 1; $se_{1}=$ standard error of item difficulty specific to subgroup 1; $d_{2}=$ item difficulty estimates specific to subgroup 2; $se_{2}=$ standard error of item difficulty specific to subgroup 2; * marks significance at .05.

**Table 6:** Calibration of item characteristics with 0.95 CI via the LLTM.

Item Characteristic	η	*SE*	Lower CI	Upper CI
Npieces	−0.85	0.29	−1.41	−0.29
Lpieces	1.20	0.25	0.71	1.68
Tedges	1.54	0.28	0.99	2.08
Medges	0.64	0.19	0.27	1.01
Cpieces	1.02	0.20	0.62	1.41
EED	−1.19	0.23	−1.65	−0.74
RSS	−0.39	0.32	−1.02	0.25
Dpieces	1.89	0.32	1.25	2.52
Rpieces	−0.44	0.22	−0.88	−0.01

Note. *SE* = standard error; CI = confidence interval; Npieces = number of pieces; Lpieces = pieces with labels; Tegdes = total edges; Medges = maximum edges; Cpieces = curved pieces; EED = easily excluded distractors; RSS = regular-shape solution; Dpieces = displaced pieces; Rpieces = rotated pieces.

**Table 7:** Item difficulty parameters ($\delta^{\prime }$) with 0.95 CIs via the LLTM.

Item	$\delta^{\prime }$	*SE*	Lower CI	Upper CI
1	−0.94	0.42	−1.77	−0.11
2	−0.94	0.42	−1.77	−0.11
3	−0.57	0.45	−1.45	0.32
4	−0.05	0.57	−1.16	1.07
5	1.32	0.44	0.45	2.18
6	0.94	0.55	−0.14	2.02
7	0.76	0.56	−0.33	1.86
8	2.04	0.55	0.97	3.11
9	0.12	0.50	−0.87	1.11
10	0.98	0.39	0.22	1.74
11	1.05	0.46	0.16	1.95
12	1.50	0.42	0.68	2.31
13	1.57	0.45	0.68	2.45
14	2.51	0.50	1.54	3.48
15	2.07	0.52	1.05	3.10

Note. *SE* = standard error; CI = confidence interval.

## Data Availability

Available on request through the third author.
